# Patient-specific dosimetry of ^99m^Tc-HYNIC-Tyr^3^-Octreotide in children

**DOI:** 10.1186/s40658-017-0191-6

**Published:** 2017-10-13

**Authors:** Xinchi Hou, Bozena Birkenfeld, Hanna Piwowarska-Bilska, Anna Celler

**Affiliations:** 1 Medical Imaging Research Group, Department of Radiology, University of British Columbia, 828 West 10th Avenue, Rm 366, Vancouver, BC V5Z1L8 Canada; 2 Nuclear Medicine Department, Pomeranian Medical University, Szczecin, Poland

**Keywords:** Pediatric patient-specific dosimetry, ^99m^Tc-HYNIC-Tyr^3^-octreotide, SPECT/CT, Neuroendocrine tumors

## Abstract

**Background:**

Technetium-99m-hydrazinonicotinamide-Tyr^3^-octreotide (^99m^Tc-HYNIC-TOC) is recognized as a promising radiopharmaceutical for diagnosing neuroendocrine tumors (NETs). However, ^99m^Tc-HYNIC-TOC dosimetry has been investigated only for adults. As pediatric radionuclide therapies become increasingly common, similar dosimetric studies for children are urgently needed. The aim of this study is to report personalized image-based biodistributions and dosimetry evaluations for children studies performed using ^99m^Tc-HYNIC-TOC and to compare them with those from adult subjects.

Eleven children/teenage patients with suspected or diagnosed NETs were enrolled. Patient imaging included a series of 2–3 whole-body planar scans and SPECT/CT performed over 2–24 h after the ^99m^Tc-HYNIC-TOC injections. The time-integrated activity coefficients (TIACs) were obtained from the hybrid planar/SPECT technique. Patient-specific doses were calculated using both the voxel-level and the organ-level approaches. Estimated children doses were compared with adults’ dosimetry.

**Results:**

Pathologic uptake was observed in five patients. TIACs for normal organs with significant uptakes, i.e., kidneys, spleen, and liver, were similar to adults’ TIACs. Using the voxel-level approach, the average organ doses for children were 0.024 ± 0.009, 0.032 ± 0.017, and 0.017 ± 0.007 mGy/MBq for the kidneys, spleen, and liver, respectively, which were 30% larger than adults’ doses. Similar values were obtained from the organ-level dosimetry when using OLINDA with adapted organ masses. Tumor doses were 0.010–0.024 mGy/MBq. However, cross-organ contributions were much larger in children than in adults, comprising about 15–40% of the total organ/tumor doses. No statistical differences were found between mean doses and dose distributions in patients with and without pathologic uptakes**.**

**Conclusion:**

Although the children TIACs were similar to those in adults, their doses were about 30% higher. No significant correlation was found between the children’s doses and their ages. However, substantial inter-patient variability in radiotracer uptake, indicating disparity in expression of somatostatin receptor between different patients, emphasizes the importance and necessity of patient-specific dosimetry for clinical studies.

## Background

Somatostatin receptor scintigraphy (SSRS) has been identified as an efficient diagnostic technique for localizing and staging neuroendocrine tumors (NETs) which overexpress somatostatin receptors [1, 2]. The benefits of using SPECT and PET for diagnostic imaging of NETs are already well recognized. Currently, the most popular tracers used for NETs diagnosis are ^99m^Tc-hydrazinonicotinamide-Tyr^3^-octreotide (^99m^Tc-HYNIC-TOC) and ^111^In-diethylenetriaminepentaacetic acid (DTPA)-octreotide for SPECT [1, 3, 4] and ^68^Ga-DOTA-conjugated peptides for PET [5–9]. Although PET/CT imaging using ^68^Ga-DOTA-conjugated peptides has been shown to have high diagnostic accuracy and low radiation exposure [10, 11], limited availability of PET cameras worldwide may restrict its clinical use. On the other hand, SPECT, and also planar scintigraphy, remain the most popular nuclear medicine imaging techniques. Considering the SPECT tracers mentioned above, ^99m^Tc-HYNIC-TOC results in better image quality and lower radiation dose than ^111^In-labeled pharmaceuticals [10, 12, 13]. Additionally, ^99m^Tc-HYNIC-TOC can be obtained using a simple, single-vial kit formulation [14] which makes it especially advantageous for use in centers with limited resources [15]. Finally, labeling with technetium makes this tracer more readily available and less expensive than tracers labeled with ^68^Ga.

The incidence of NETs is rather low in the adult population and even lower in children. Recently, however, its rate is steadily increasing [16–18]. The SSRS studies are often used not only for the initial diagnosis and localization of the disease, but also for the therapy follow-up or diagnosis of the disease recurrence. Hence, information about radiation exposure, be it due to single administration or to repeated procedures, is very important, especially in children.

Since ^99m^Tc-HYNIC-TOC diagnostic studies provide information about this radiotracer uptake and biodistribution, evaluation of its uptake in organs at risk (OAR) can predict potential toxicities. At the same time, this information may be used in radionuclide therapy planning, because its uptake in tumors is proportional to the intensity of somatostatin receptor expression in tumor cells, thus it may indicate potential effectiveness of the treatment.

Additionally, using the theranostic approach, the dose calculated for diagnostic isotope can be used to predict that which will be delivered when the diagnostic agent is replaced by the therapeutic one, (labeled with a beta-emitting radioisotope). These considerations will apply to the ^99m^Tc-HYNIC-TOC imaging studies. Quantification of its uptake and biodistribution in tumors and OARs can be used in ^99m^Tc-HYNIC-TOC dosimetry calculations and at the next stage to predict doses which will be delivered by peptide receptor therapy agents such as DOTA-TATE or DOTA-TOC labeled with ^177^Lu or ^90^Y. Although this procedure involves pharmaceuticals which are not identical, recent studies comparing pre-therapy biodistributions of ^99m^Tc-HYNIC-TOC and ^68^Ge-DOTA-TATE with that of post-therapy of ^177^Lu-DOTA-octreotate support the validity of this approach [15].

Currently, only limited radiation dosimetry information is available for ^99m^Tc-HYNIC-TOC. The earliest dosimetry study, published in 2006 by Gonzalez-Vazquez et al., determined absorbed radiation doses for eight patients using a two-dimensional dosimetry protocol based on five whole-body scans [19]. In 2011, our group reported patient-specific dosimetry calculations for ^99m^Tc-HYNIC-TOC based on a larger study of 28 subjects [20]. A hybrid imaging protocol, including single SPECT/CT combined with a series of whole-body planar scans was used. Additionally, a 3D Monte Carlo dose estimation has been published by Momennezhad et al. in 2016 [21].

All of these dosimetry studies were done only for adults. However, the absorbed dose estimates are especially important for children [18, 22]. This is because children have an increased risk of potential adverse health effects due to their higher sensitivity to radiation and longer life expectancy resulting in increased opportunity for experiencing radiation-related cancers or cardiovascular and other non-cancer diseases [23]. Additionally, due to their smaller body sizes and potential differences in radiotracer uptakes, dosimetry calculations performed for adult subjects may not be appropriate for children. Finally, when considering the adult population, multiple studies have shown significant dose differences between individual subjects [20]. Thus, similar patient-specific dosimetry studies are needed for children.

For these reasons, the importance of dosimetry for pediatric radionuclide diagnostic and therapeutic procedures [24, 25] and also for CT imaging [26] cannot be underestimated. Currently, however, pediatric dosimetry calculations are limited to only a few child models (OLINDA software allows only for dosimetry calculation for newborn, 5, 10, and 15 years old) [27, 28].

The objective of the present study is to improve this situation. To this end, we applied the same methods as were used in our personalized image-based dosimetry determination for ^99m^Tc-HYNIC-TOC imaging studies in adult subjects, to process and analyze 12 datasets from children’s studies. A hybrid planar/SPECT technique was employed to estimate the cumulated activities and biodistributions [20]. Both voxel-level (voxel-S) and organ-level dosimetry approaches were used to evaluate tumors’ and normal organs’ doses. Comparison between children doses and those estimated for adults [20] was performed.

## Methods

### Patient studies

Eleven children/teenage patients (ages 2–17 years, 4 males and 7 females) with suspected or diagnosed NETs were enrolled into this investigation, and a total of twelve imaging studies were performed. Table 1 lists these patients’ demographic information. All the patient imaging studies were performed at the Nuclear Medicine Department, Pomeranian Medical University, Szczecin, Poland.

**Table 1:** Demographic information of children patients involved in this study, sorted by age

Patient #	Sex	Age(year)	Injected activity (MBq)	Diagnosis
1	M	2	320	Mediastinum 7-cm diameter NET
2	F	7	450	
3	M	11	490	Right adrenal pheochromocytoma (only shown in SPECT/CT image)
4-A^a^	F	12	500	
5	F	12	600	
4-B^a^	F	13	450	
6	F	13	480	Appendix NET, area of accumulation above the uterus
7	F	14	600	MEN-1, head of the pancreas NET and lymph node accumulation on the left side
8	M	14	940	
9	F	16	500	
10	F	16	600	
11	M	17	720	MEN-1, head of the pancreas insulinoma

^a^Patient #4 had two scans done in the interval of one year, i.e., 4-A and 4-B

The injected activities ranged from 320 to 940 MBq. For each patient, a series of two or three whole-body planar scans and a single SPECT/CT scan were performed within the 24-h time period after the injection. The majority of studies used a dual-head Infinia Hawkeye4 camera (GE Healthcare), while three whole-body planar scans were performed using a Nucline X-Ring/R camera (Mediso Medical Imaging Systems). For the SPECT/CT scans, 60 projections with 20 s/projection acquisition time were collected over the 360° non-circular orbit. The projection matrices of SPECT scans were 128 × 128 with 4.418-mm pixel size, while the matrices used for planar scans were 256 × 1024 with 2.21-mm pixel size or 512 × 1024 with 2.88-mm pixel size. A low-dose CT was used to generate attenuation maps. Additionally, for one patient, a 30-min dynamic scan was performed to study pharmacokinetics of the radiopharmaceutical uptake phase.

### SPECT image reconstruction and quantification

SPECT images were reconstructed using the camera software with iterative ordered-subsets expectation maximization algorithm (OSEM) with 2 iterations and 10 subsets. The CT-based attenuation correction and Hann filter were included in the reconstruction. Although no scatter correction was applied, the attenuation map was rescaled to the broad beam values which indirectly corrected for scatter.

In order to achieve accurate image quantification, camera calibration, necessary to convert counts in the reconstructed image to activity, was performed using a point source and a planar acquisition. A 3-mL vial filled with 4.82 MBq of ^99m^Tc was placed in air centrally between the two detectors. The camera sensitivity factor was determined by averaging the value from the two detectors.

### Pharmacokinetics calculations

For use in dosimetry calculations based on the hybrid planar/SPECT approach, first, the time-activity curves (TACs) for all organs with significant uptakes (kidneys, liver, spleen) and tumors were determined from a series of whole-body (WB) planar scans. The following segmentation method was used. For each of these organs/tumors, an oversized region of interest (ROI) was manually drawn in the first WB image (acquired < 3 h after injection). A threshold set at 50% of a maximum pixel count in this ROI was then used to create a smaller 2D ROI_50_. Subsequently, this 2D ROI_50_ was registered to the entire sequence of the WB scans. Additionally, in order to perform geometrically based background subtraction [20, 29], a small ROI in the background region adjacent to each investigated organ was drawn. For each ROI_50_, the TAC was generated by plotting the background corrected mean-counts in this 2D ROI_50_ versus scan-time (hours after injection) and then fitting a mono-exponential curve to this data. From this procedure, the organ and tumor TACs, expressed in relative units of counts per hour, were obtained. Then, the effective and biological half-lives for each organ were determined.

**Fig. 1:**
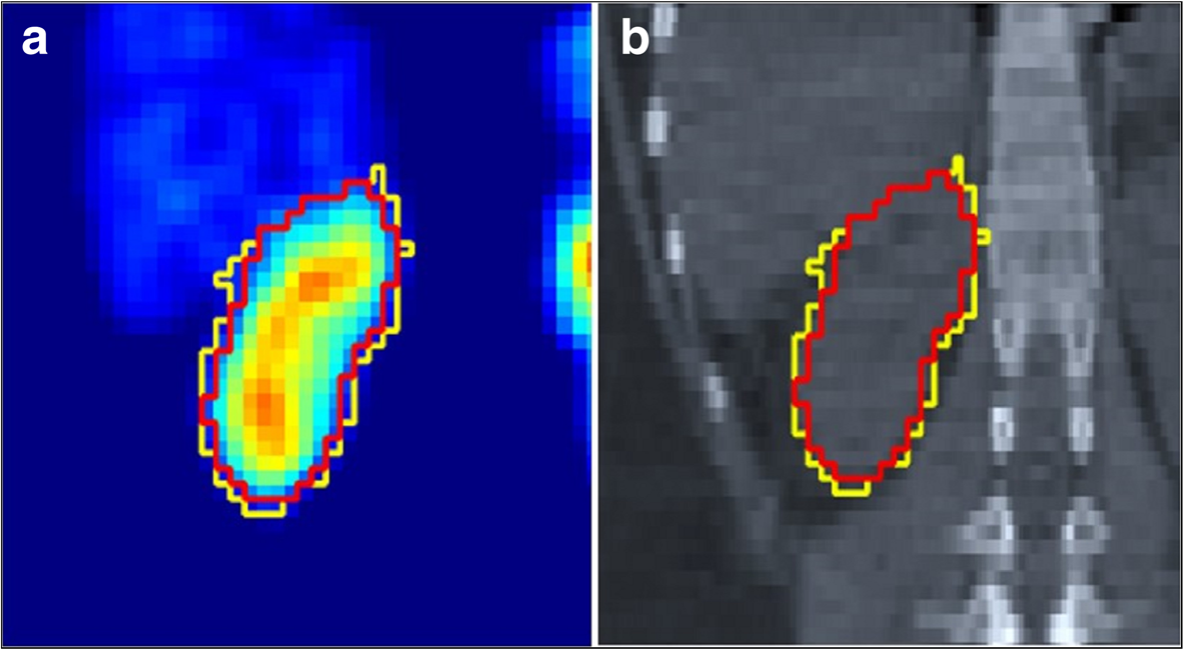
An example of the right kidney segmentation based on dual iterative thresholding method in SPECT (**a**) and CT (**b**). The activity threshold was set to 25% (yellow line), and the volume threshold was equal to 36% (red line)

At the next stage, the time-integrated activity coefficients (TIACs) were generated by rescaling these TACs using absolute organ activities obtained from the quantitatively reconstructed SPECT/CT images. For each organ and tumor, our dual iterative adaptive thresholding method [20, 30] was used to determine this organ/tumor volume and activity. This method uses a series of phantom experiments to generate two calibration curves. They provide the optimal thresholds, one for determining the true volume and the other for determining the true activity of the hot object placed in a warm background, as a function of the observed signal-to-background ratio of this object. The value of the threshold depends on the image reconstruction method, thus it indirectly accounts for partial volume effects and, for objects larger than 12 mL, has been shown to not depend on the object size [30]. An example of kidney segmentation using the dual iterative adaptive thresholding method is shown in Fig. 1.

### Dosimetry calculations

For each investigated organ and tumor, its mean dose and dose distribution (represented by the cumulated dose-volume histograms—DVHs) were estimated using the voxel-S approach. Similar to our previous dosimetry study of adult patients, the analysis of images and the dose calculations were performed using our in-house developed dosimetry software JADA [31]. The matrices of voxel-S value were pre-calculated with the EGSnr DOSXYZnrc Monte Carlo program. The voxel array corresponded to a 215 × 215 × 215 grid with voxel size of 4.418 mm, which was large enough for estimation of the cross-organ dose exchange between all the ROIs. More details about this approach can be found in [32]. Using this method, both self- and cross-organ doses were studied.

In parallel, the OLINDA 1.1 [28] dosimetry software was employed for organ-level dosimetry. The TIACs for the main organs, i.e., kidneys, liver, and spleen, were determined using the JADA software, whereas TIACs for the urinary bladder contents were calculated using the voiding bladder model provided by OLINDA. Additionally, the TIACs for the remainder of the body were estimated by subtracting all TIACs of the investigated organs from those corresponding to the whole body. Since OLINDA 1.1 provides only models for children 1, 5, 10, and 15 years old, for each of our patients, the calculations were performed using the model which was closest to his/her age. Organ-level dosimetry obtained from OLINDA with and without adjusted organ masses from patients’ SPECT/CT images were studied. The doses for tumors were estimated based on the OLINDA spherical model.

Finally, the dose estimated using the two methods described above were compared with each other and with the adult doses derived from our previous study [20]. Furthermore, the relationship between the children’s doses and their ages was analyzed.

## Results

### TIAC and effective half-life

**Fig. 2:**
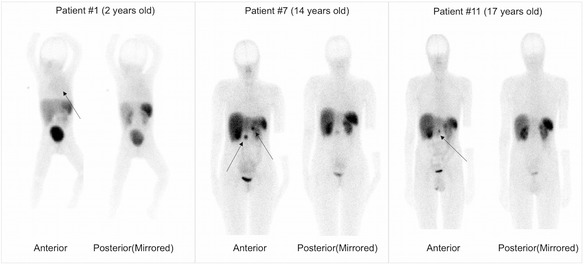
Whole-body planar images acquired at approximately 1.5–2.5 h after injection, with pathologic uptake indicated by arrows. Patient #1: a large tumor with low uptake located in the mediastinum; patient #7: two tumors, one located in the pancreatic head and the other in the lymph node on the left side; patient #11: a single tumor located at the center of the patient’s body (insulinoma head of pancreas)

In patient imaging studies, six tumors were discovered in five of the patients, as shown in Table 1. Fig. 2 displays examples of the whole-body images acquired within 3 h after injection from three of the patients who had visible tumors. The locations of tumors are indicated by arrows in this figure. The main uptake organs were the kidneys, liver, and spleen.

**Fig. 3:**
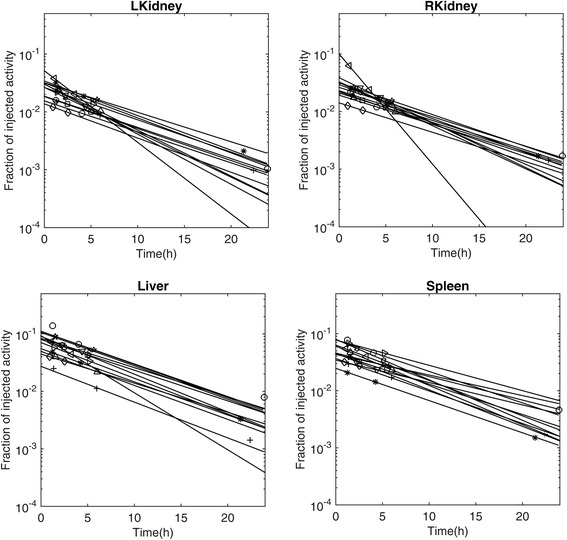
Organ time-activity curves based on mono-exponential fittings for all of the patients. Different symbols correspond to different patients

**Fig. 4:**
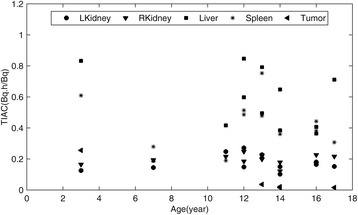
Organ and tumor TIACs displayed versus patient age. Symbols represent different organs and tumors, including the left and right kidneys, liver, spleen, and tumor

The time-activity data for the left and right kidneys, the liver, and the spleen are displayed in Fig. 3 together with the curves corresponding to the mono-exponential fit to this data. The mean effective and biological half-lives determined from these time-activity curves are summarized in Table 2 for normal organs and tumors. Furthermore, the relationship between these TIACs and the ages of the patients are depicted in Fig. 4. The mean values of the children’s TIACs for the analyzed organs and tumors, together with the adults’ TIACs, are summarized in Table 3. Please note that all the data shown in Tables 2, 3, and 4 correspond to the average value ± standard deviation; the ranges of these average values are shown in parentheses.

**Table 2:** Effective and biological half-lives determined from the mono-exponential fits to the data of radiotracer uptakes in organs and lesions in children patients

Organ	Effective half-life (hour)	Biological half-life (hour)
LKidney	4.72 ± 1.08 (2.44–5.93)	69.5 ± 117.1 (4.1–431.4)
RKidney	4.94 ± 1.32 (1.57–5.99)	308.5 ± 534.2 (2.1–1873.3)
Liver	5.07 ± 0.76 (3.04–5.99)	182.4 ± 429.6 (6.1–1521.6)
Spleen^a^	5.83 ± 1.33 (4.26–8.22)	15.0 ± 42.8 (− 54.8–90.5)
Tumors	4.18 ± 1.21 (2.86–5.71)	35.0 ± 45.7 (5.4–112.9)

^a^The effective half-life of spleens in four of the patients were higher than ^99m^Tc physical half-life

**Table 3:** Comparison of organ TIAC for children to those for adults

Organ	TIAC (Bq.h/Bq)	
	Children	Adults [20]
Kidneys^a^	0.37 ± 0.08 (0.22–0.52)	0.35 ± 0.10 (0.19–0.54)
Liver	0.56 ± 0.21 (0.19–0.85)	0.75 ± 0.31 (0.20–1.72)
Spleen	0.43 ± 0.15 (0.19–0.75)	0.43 ± 0.20 (0.04–0.89)
Urinary bladder^b^	0.17 ± 0.19 (0.05–0.28)	0.23 ± 0.09 (0.12–0.53)
Remainder of body^c^	5.01 ± 2.15 (2.52–8.92)	4.34 ± 0.74 (3.00–6.10)
Tumors	0.07 ± 0.10 (0.02–0.26)	–

^a^The TIAC of the kidneys were obtained by performing the time-activity curve fitting for both the left and right kidneys

^b^The TIAC values of urinary bladder contents were calculated using the voiding bladder model provided by OLINDA

^c^The TIACs for the remainder of the body were estimated by subtracting the organs’ TIACs from the whole-body TIACs

**Table 4:** Comparison of absorbed doses in organs and tumors in children and adults

Organ	Mean dose (mGy/MBq)		
	Children (Voxel-S approach)	Children (OLINDA with adapted organ masses)	Adults (OLINDA with adapted organ masses) [20]
Kidneys	0.024 ± 0.009 (0.011–0.047)	0.026 ± 0.009 (0.009–0.046)	0.021 ± 0.007 (0.011–0.039)
Spleen	0.032 ± 0.017 (0.019–0.081)	0.038 ± 0.021 (0.017–0.092)	0.030 ± 0.012 (0.005–0.057)
Liver	0.017 ± 0.007 (0.009–0.032)	0.016 ± 0.007 (0.007–0.029)	0.012 ± 0.005 (0.005–0.028)
Tumor^a^	0.017 ± 0.006 (0.010–0.024)	0.010 ± 0.003 (0.007–0.015)	0.024 ± 0.016 (0.003–0.047)

^a^Tumor masses are 14–546 g for children, while 8–855 g from adults

**Fig. 5:**
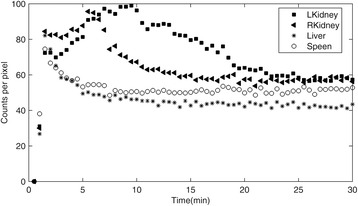
Time-activity curves showing radiotracer uptake in the kidneys, liver, and spleen. The data were obtained from a 30-min dynamic planar scan

In order to investigate the uptake phase of ^99m^Tc-HYNIC-TOC pharmacokinetics, a 30-min dynamic planar scan with a total of 60 time frames was performed for one patient. For the plotting of the changes of tracer uptake versus time, a threshold of 50% of the maximum pixel counts in each region was applied to each time frame and the counts were summed in the segmented regions. The resulting curves are shown in Fig. 5.

### Absorbed doses

**Fig. 6:**
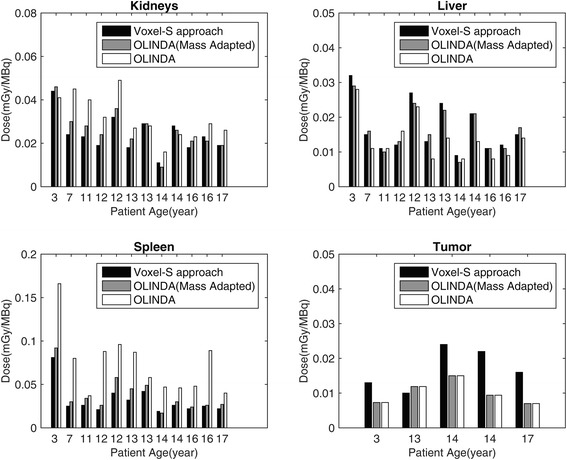
Absorbed doses in the organs and tumors calculated using the voxel-S approach, the OLINDA with adapted organ masses and OLINDA with default organ masses displayed versus patient age

**Fig. 7:**
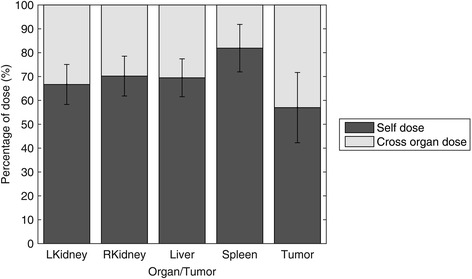
Average percent contributions of self- and cross-organ doses to the total organ and tumor doses for children patients, calculated using voxel-S approach. Error bars represent standard deviations estimated from all patients’ data

**Fig. 8:**
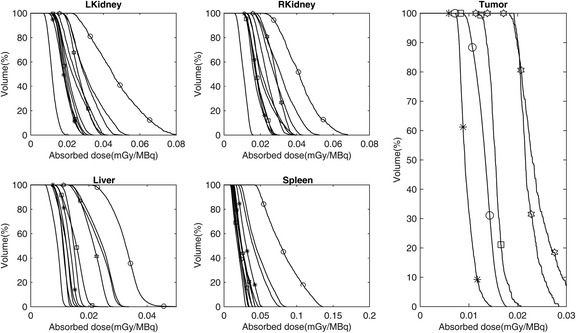
Dose-volume histograms (DVHs) for normal organs and tumors. Each line represents a different patient; the lines with symbols represents the patients with pathologic uptakes

Figure 6 displays the absorbed doses in children’s organs and tumors estimated using the voxel-S approach (by convolving voxel-S matrices with the activity distributions) and the organ-level method (from OLINDA software with/without adapted organ masses). The mean doses are summarized and compared with the adults’ dosimetry in Table 4. Fig. 7 displays the percent contributions of self- and cross-organ doses to the total organ and tumor doses for children patients, calculated using the voxel-S approach. Additionally, dose distributions, in terms of the cumulated DVHs, are presented in Fig. 8.

## Discussion

In this study, the investigation of the biodistribution of ^99m^Tc-HYNIC-TOC in children patients and the corresponding dosimetry calculations were performed and compared with those of adults. The study employed the hybrid planar/SPECT imaging technique. The main radiopharmaceutical uptake occurred in the kidneys, liver, and spleen. Although in our previous adult study some thyroid uptake was observed [20], no significant uptake was found in the children (shown in Fig. 2).

The TACs and TIACs determined for the kidneys, liver, and spleen are shown in Fig. 3 and Table 3. The average values of organ TIACs were 0.37 ± 0.08, 0.56 ± 0.21, and 0.43 ± 0.15 for the kidneys, liver, and spleen, respectively. The children’s TIACs for the kidneys and spleens were very similar to those obtained in the adult’s study, while TIACs for the livers in children patients were smaller than those in adults [20].

Dynamics of the radiotracer uptake was investigated in one of the patients. The analysis of this temporal behavior, displayed in Fig. 5, showed maximum uptake for the kidneys at about 7–10 min, while faster maximum uptake happened, at about 1–3 min, for both the liver and spleen. After reaching their maximum uptake, a washout phase was observed for both the kidneys and livers. However, for the spleen, after a rapid decrease following the peak uptake, a gradually increasing uptake seemed to continue beyond the time covered by this 30-min dynamic scan. This could explain the negative biological half-lives found in four of the patients, as indicated in Table 2.

Considering dosimetry for normal organs, as shown in Table 4 and Fig. 6, no significant differences were found between voxel-level dosimetry and organ-level dosimetry when using adapted organ masses in OLINDA for the kidneys and livers. On the other hand, the voxel-level doses for the spleen were slightly lower than organ-based doses. However, for all the normal organs, large discrepancies could be observed between voxel-level doses and those calculated using OLINDA without adapting organ masses. This indicates that dosimetry using OLINDA with default organ masses, especially for children models, may not reflect their true absorbed doses.

The tumor doses (shown in Table 4, Fig. 6, and Fig. 7) from the spherical model obtained using OLINDA were approximately equal to only half of the voxel-level tumor doses. This discrepancy is related to the fact that the spherical model only allows for calculation of the self-tumor doses; however, for tumors located close to the organs with large uptakes (like the kidneys, liver, or spleen), the cross-organ contributions may be substantial. This effect can be especially pronounced in small children, where distances between organs are smaller than in adults. It is more important in dose estimates for photons, because they deposit their energy at distances larger than, for example, beta particles, whose dose depositions are more localized.

When comparing children dosimetry to that for adults (listed in Table 4), organ doses for children were found to be up to 30% higher than those for adults, due to smaller organ sizes. This effect is related to the fact that, as already noted, the children’s and adults’ TIACs were similar, while the absorbed doses are inversely proportional to the organ masses. However, the tumor doses for children were lower than those for adults. Additionally, as shown in Fig. 7, children’s cross-organ contributions were equal to about 15–40% of the total organ and tumor doses, while for adults, the cross-organ doses were less than 15% of the total doses (see Fig. 3a in [32]).

Furthermore, no statistical correlation was noted between TIACs (Fig. 4), the absorbed doses (Fig. 6), and the age of children (please note, however, this statement is based on the analysis of a small sample of only 11 patients). Similarly, no statistical differences were found between mean doses and dose distributions (represented by DVHs) in patients with and without pathologic uptakes. However, large inter-patient variability was clearly observed (Fig. 6 and Fig. 8), which emphasizes the importance of performing patient-specific dosimetry in clinical studies.

## Conclusions

In this study, we report the results of biodistribution analysis and dosimetry calculations for children who had ^99m^Tc-HYNIC-TOC injections for the diagnosis of neuroendocrine tumors. The absorbed doses in children were slightly higher than those in adults. No significant correlation was found between the children’s doses and their ages. However, substantial inter-patient variability in radiotracer uptake, potentially indicating disparity in expression of somatostatin receptors between different patients, emphasizes the importance and necessity of patient-specific dosimetry in future clinical studies.

## Abbreviations

^99m^Tc-HYNIC-TOC: Technetium-99m-hydrazinonicotinamide-Tyr^3^-octreotide
DVH: Dose-volume histogram
NET: Neuroendocrine tumor
OAR: Organs at risk
OSEM: Ordered-subsets expectation maximization
ROI: Region of interest
SSRS: Somatostatin receptor scintigraphy
TAC: Time-activity curve
TIAC: Time-integrated activity coefficient
WB: Whole-body
